# Virtual mapping of 260 three-dimensional hemipelvises to analyse gender-specific differences in minimally invasive retrograde lag screw placement in the posterior acetabular column using the anterior pelvic and midsagittal plane as reference

**DOI:** 10.1186/s12891-015-0697-9

**Published:** 2015-09-04

**Authors:** Bjoern Gunnar Ochs, Fabian Maria Stuby, Ulrich Stoeckle, Christoph Emanuel Gonser

**Affiliations:** BG Trauma Centre, Eberhard Karls University of Tuebingen, Schnarrenbergstraße 95, 72076 Tuebingen, Germany

## Abstract

**Background:**

Due to complex pelvic geometry, percutaneous screw placement in the posterior acetabular column can pose a major challenge even for experienced surgeons.

**Methods:**

The present study examined the preformed bone stock of the posterior acetabular column in 260 hemipelvises. Retrograde posterior column screws were virtually implanted using iPlan® CMF (BrainLAB AG, Feldkirchen, Germany); maximal implant length, maximal implant diameter and angles between the screw trajectories and the reference planes anterior pelvic plane as well as the midsagittal plane were assessed for gender-specific differences.

**Results:**

The virtual analysis of the preformed bone stock column showed two constrictions of crucial clinical importance. These were located 49.6 ± 3.4 (41.0–60.2) mm (inferior margin of acetabulum) and 77.0 ± 5.6 (66.5–95.3) mm (centre of acetabulum) from the entry point of the implant in men and respectively 43.7 ± 2.3 (38.3–49.3) mm as well as 71.2 ± 3.5 (63.5–79.99) mm in women (men vs. women: *p <* 0.001). The entry point of the retrograde posterior column screw was located dorsal from the transition of the lower margin of the ischial tuberosity to ramus inferior pointing to the medial margin of the ischial tuberosity. In female patients, the entry point was located significantly closer to the medial margin of the ischial tuberosity. However, 7.3 mm screws can generally be used in men and women. The angle between the screw trajectory and the anterior pelvic plane in sagittal section was 14.0 ± 4.9 (2.5–28.6) °, the angle between the screw trajectory and the midsagittal plane in axial section was 31.1 ± 12.8 (1.5–77.9) ° and the angle between the screw trajectory and the midsagittal plane in coronal section was 8.4 ± 3.8 (1.5–20.0) °. For all angles, significant gender-specific differences were found (*p <* 0.001).

**Conclusion:**

Therefore, the anterior pelvic plane as well as the midsagittal plane can facilitate intraoperative orientation for retrograde posterior column screw placement considering gender-specific differences in preformed bone corridor, implant length as well as angles formed between screw trajectory and these reference planes.

## Background

Anatomic reduction and retention are pivotal in surgical therapy of acetabular fractures to prevent posttraumatic osteoarthritis. Open reduction and stabilization via ilioinguinal or Kocher-Langenbeck-approach and in rare cases extended approaches is the internationally accepted standard [1].

Minimally-invasive osteosynthesis approaches are limited due to the demanding reduction, which cannot always be completely achieved, and because of the complex three-dimensional geometry of the pelvis with limited bone corridors for screw placement. Fluoroscopy-assisted percutaneous screw osteosynthesis of the acetabulum may require several trials for correct screw placement which leads to high radiation doses [2].

Based on previous findings [3, 4] suggesting that there are gender-specific differences regarding the best implant position, we first defined an optimal screw position for the retrograde placed posterior column screw and then analysed the surrounding preformed bone corridor. In order to gain statistically relevant data without the limitation of a reduced sample size of previously performed studies [3, 5–8], we decided to perform a virtual study using the technique described in 2010 [2]. We examined 260 hemipelvises virtually using data from CT scans of 130 uninjured European pelvises which were collected during clinical routine exams at our trauma centre. Our first hypothesis was that there are gender-specific differences considering the optimal implant position.

In a second step, we wanted to analyse, if there are landmark pelvic planes which could be used as reference, thus facilitating the visualization and screw placement for the surgeon. We have chosen the anterior pelvic plane as well as the midsagittal pelvic plane as reference planes since they are commonly used in hip arthroplasty [9]. We analysed the angle between the screw trajectory and these planes. Our second hypothesis was, that there are gender-specific differences considering the angles between these planes and the optimal screw position.

## Methods

This study has been approved by the ethical committee of the University of Tuebingen (No 580/2012R). A patient collective of 260 uninjured hemipelvises was examined in regard to significant gender-specific differences relevant in the placement of the posterior column screw. Therefore, a CT-scan archive spanning from 06/2003 till 06/2010 was searched for exams of the pelvis to a standardized protocol (16 line helical CT Siemens Somatom Sensation 16, 120 kV, 160 mAs, slice thickness of 0.75 mm, collimation 0.75; Siemens AG, Munich, Germany). Patients with fractures of the acetabulum or pelvic ring, hip dysplasia or advanced osteoarthritis of the hip and age < 16 years or > 86 years were excluded as well as patients who underwent arthroplasty of the hip or osteosynthesis of the pelvic ring or acetabulum. Moreover, the study included only European patients.

### Patient collective

The analysis of anatomically preformed periacetabular screw corridors was performed on 130 CT-Scans of uninjured Europeans (260 hemipelvises; 65 male/65 female patients). The collective showed a medium age of 50.9 ± 20.3 (16–85) years and a medium BMI of 25.8 ± 5.3 (18.2–46.1) kg/m^2^ (Table 1). Written informed consent for participation in the study was obtained from all participants.

**Table 1:** Patient collective. Age, body length and weight of examined Europeans with uninjured pelvis

Patient collective: *N =* 130				
	Age	Length (m)	Weight (kg)	BMI (kg/m^2^)
Mean ± SD	50.9 ± 20.5	1.71 ± 0.1	75.3 ± 16.6	25.8 ± 5.3
Median	52.0	1.72	74.0	25.3
Range	16–85	1.50–1.92	42–134	18.2–46.1
Men: *N =* 65				
	Age	Length (m)	Weight (kg)	BMI (kg/m^2^)
Mean ± SD	49.7 ± 20.2	1.76 ± 0.1	79.9 ± 14.4	25.9 ± 4.6
Median	51.0	1.75	77.0	25.5
Range	17–85	1.60–1.92	55–134	19.5–43.3
Women: *N =* 65				
	Age	Length (m)	Weight (kg)	BMI (kg/m^2^)
Mean ± SD	52.1 ± 20.8	1.65 ± 0.1	69.8 ± 17.5	25.6 ± 6.2
Median	53.0	1.65	65.6	24.2
Range	16–85	1.50–1.78	42–130	18.2–46.1

Statistical analysis revealed that there was no significant differences between female and male patients considering age (*p =* 0.548) and BMI (*p =* 0.742). However men were significantly taller (*p <* 0.001) and heavier (*p =* 0.006) than women.

### Analysis of anatomical preformed bone corridor for the posterior column screw

**Fig. 1:**
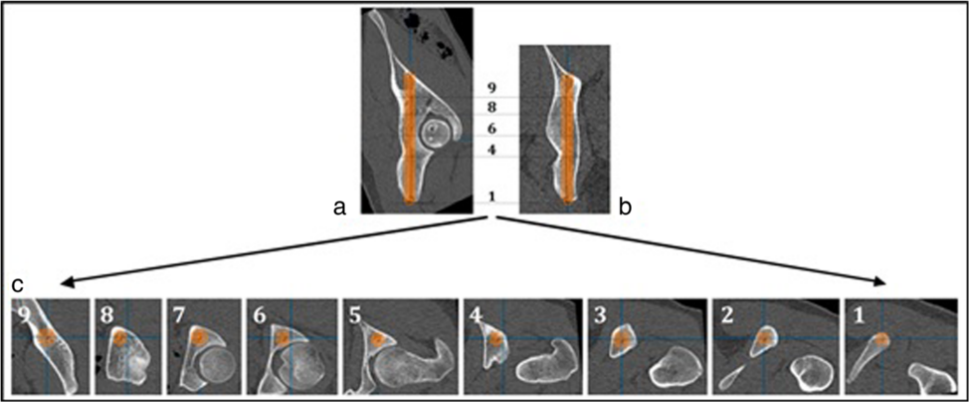
Planning mask for the posterior column screw. Planning mask for the posterior column screw resembling the anatomical bone corridor of the posterior column in regard to the optimal screw position. Fig. 1**a** shows a sagittal, Fig. 1**b** a coronal section. Fig. 1**c** shows nine axial sections which were planned according to Ebraheim et al. [5]. (*8*) axial section at the upper acetabular margin (*6*) axial section at the centre of acetabulum and (*4*) at the lower margin of acetabulum (*9*) axial section in the middle of the stretch between screw exit point and upper acetabular margin (*7*) axial section in the middle of the stretch between upper acetabular margin and centre of the acetabulum (*5*) axial section in the middle of the stretch between centre of acetabulum and lower acetabular margin (*3*) axial section at two thirds of the stretch between screw entry point and lower acetabular margin (*2*) axial section at one third of the stretch between screw entry point and lower acetabular margin (*1*) axial section at the screw entry point. In each of the axial sections, the maximum screw diameter is shown

The analysis of the individual anatomical preformed corridor for the screw was performed on 260 hemipelvises. First, the maximal implant length and the maximal implant diameter were assessed. Therefore, the screw trajectory was increased in diameter until the implant reached the inner corticalis without penetrating it. Maximal screw length was reached when the end of the virtual screw reached the corticalis of the iliac fossa. Then, according to Ebraheim et al. [6], nine (9) parallel sections—with respect to the individual anatomy—orthogonal to the screw axis were chosen and the diameter of the bone corridor and the maximal implant diameter were determined in each section. The sectional planes were positioned according to the individual anatomy of the patient and screw length to achieve intra—and interindividual comparability (Fig. 1). The entry points were defined in respect to the anatomical landmarks—ischial tuberosity and the caudal margin of the ramus inferior of ossis pubis.

### Virtual screw placement and position analysis

**Fig. 2:**
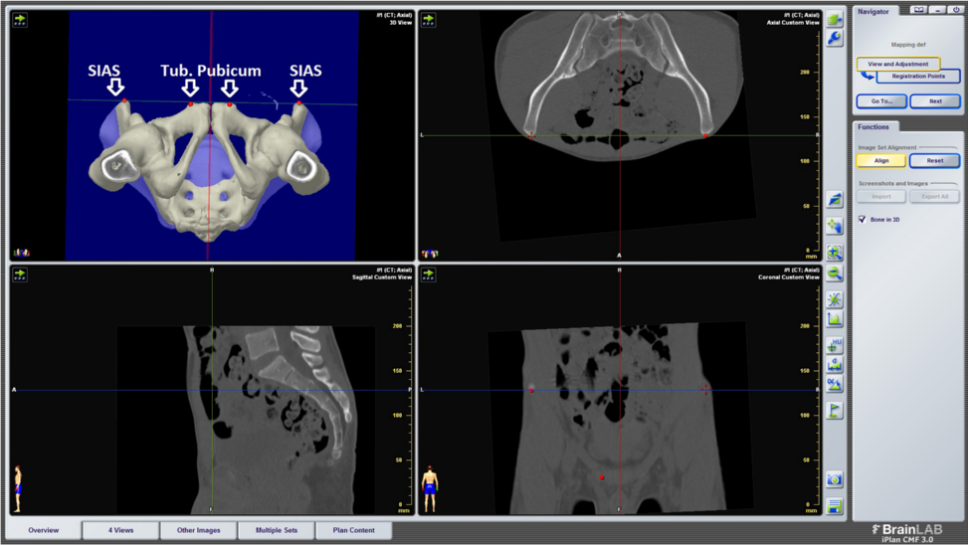
Process of placing anterior pelvic plane and midsagittal plane in the virtualized pelvis using the landmarks superior iliac anterior spine [SIAS] and tuberculum pubicum of both sides

The virtual screw placement as well as position analysis was performed using iPlan® software (BrainLab AG, Feldkirchen, Germany). To calibrate the software and to set up the desired planes, several landmarks of the pelvis had to be manually registered. These landmarks included the anterior superior iliac spine on both sides as well as the tuberculum pubicum on both sides. With these four points the anterior pelvic plane was determined (Fig. 2). The midsagittal plane was placed vertically to the anterior pelvic plane and through the centre of the symphysis and the centre of the sacral spine.

**Fig. 3:**
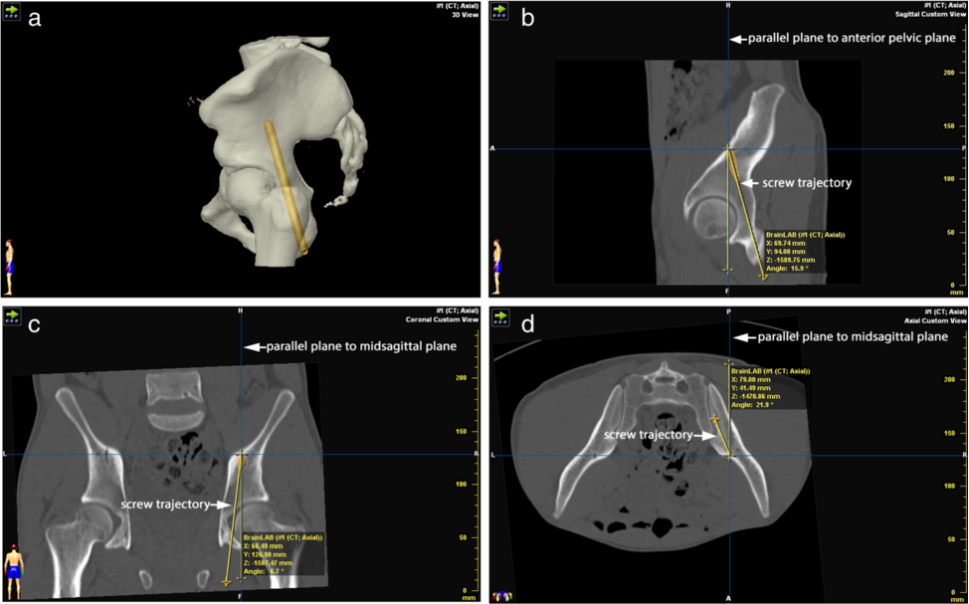
**a**: Screw trajectory (yellow) in 3D view. **b**: Posterior column screw trajectory (yellow line) and parallel to anterior pelvic plane (vertical blue line) in sagittal section forming the angle measured with embedded software module. **c**: Posterior column screw trajectory (yellow line) and parallel plane to midsagittal pelvic plane (vertical blue line) in coronal section forming the angle measured with embedded software module. **d**: Posterior column screw trajectory (yellow line) and parallel plane to midsagittal plane (vertical blue line) in axial section forming the angle measured with embedded software module

Then, the angle between the screw axis and the both pelvic planes mentioned above was measured using also iPlan® 3.0 software. This was done in sagittal sections for the anterior pelvic plane and in axial as well as coronal sections for the midsagittal plane. To use the angle measurement tool of iPlan® 3.0, a parallel plane to the respective plane was placed through the endpoint of the screw. The angle formed between this parallel plane and the screw was then measured. An example is shown in Fig. 3a-d.

### Statistical analysis

Statistical analysis was performed with IBM SPSS Statistical package Version 22. Student’s *t*-test as well as Mann–Whitney *U*-test was used to analyse the anthropometric data. Correlation analysis was performed with Pearson’s test for linear and Spearmen’s test for non-linear correlation. A p value of <0.05 was considered significant. Power analysis performed with an alpha-value of 0.05 for each test showed a power of 0.91 up to 0.98 for the sample size of 260 hemipelvises.

## Results

### Implant length and-diameter

Mean implant length in male patients was 141.8 ± 7.7 (125.8–162.0) mm and 127.2 ± 6.8 (111.7–145.3) mm in female patients (male vs. female patients: *p <* 0.001) without significant side differences in both groups (men: *p =* 0.370; women: *p =* 0.429) (Table 2). The maximal implant diameter reached 12.9 ± 1.5 (10.0–17.0) mm in men and 11.2 ± 1.3 (8.0–15.0) mm in women (men vs. women: *p <* 0.001) showing no significant side differences in both sexes (men: *p =* 0.153; women: *p =* 0.297) (Table 3).

**Table 2:** Implant length of the posterior column screw. Implant length in mm for the posterior column screw in examined Europeans with uninjured pelvis

	Implant length [mm]		
	Right	Left	Both sides
Men:			
Number	65	65	130
Mean ± SD	141.8 ± 7.6	141.9 ± 7.9	141.8 ± 7.7
Median	141.7	141.2	141.6
Range	125.8–162.0	127.3–162.0	125.8–162.0
Women:			
Number	65	65	130
Mean ± SD	127.2 ± 6.7	127.3 ± 7.0	127.2 ± 6.8
Median	126.6	126.4	126.5
Range	114.3–144.3	111.7–145.3	111.7–145.3

**Table 3:** Implant diameter of the posterior column screw. Maximum implant diameter in mm for the posterior column screw in examined Europeans with uninjured pelvis

	Implant diameter [mm]		
	Right	Left	Both sides
Men:			
Number	65	65	130
Mean ± SD	12.9 ± 1.5	13.0 ± 1.6	12.9 ± 1.5
Median	13.0	13.0	13.0
Range	10.0–16.0	10.0–17.0	10.0–17.0
Women:			
Number	65	65	130
Mean ± SD	11.3 ± 1.3	11.2 ± 1.3	11.2 ± 1.3
Median	11.0	11.0	11.0
Range	8.0–15.0	8.0–15.0	8.0–15.0

### Entry point of the posterior column screw

The entry point of the posterior column screw was located 12.7 ± 2.0 (6.6–18.1) mm dorsal from the transition of ramus pubis inferior and ischial tuberosity in anterior-posterior direction and 12.1 ± 1.9 (8.1–18.2) mm lateral of medial margin of ischial tuberosity in male patients (patient in supine position).

In female patients, the entry point was located significantly more medial (meaning closer to medial margin of the ischial tuberosity) than in male patients (10.8 ± 1.8 (8.1–18.2) mm; *p <* 0.001) (see Table 4). The patient collective (260 hemipelvises) showed a significant correlation between increasing implant diameter and increasing distance of the entry point from medial margin of ischial tuberosity (r = 0.4057).

**Table 4:** Entry Point of the posterior column screw. Entry point of the retrograde posterior column screw in relation to the transition of Ramus pubis inferior and tuber ischiadicum and medial margin of tuber ischiadicum in examined Europeans with uninjured pelvis

	Distance to the transition of Ramus pubis inferior and tuber ischiadicum [mm]			Distance to medial margin of tuber ischiadicum [mm]		
	Right	Left	Both sides	Right	Left	Both sides
Men:						
Number	65	65	130	65	65	130
Mean ± SD	12.6 ± 2.0	12.8 ± 2.0	12.7 ± 2.0	12.3 ± 1.8	11.9 ± 2.0	12.1 ± 1.9
Median	12.7	12.9	12.8	12.2	11.9	12.0
Range	6.6–18.1	8.0–17.9	6.6–18.1	8.3–16.0	8.1–18.2	8.1–18.2
Women:						
Number	65	65	130	65	65	130
Mean ± SD	12.5 ± 1.6	12.3 ± 1.6	12.4 ± 1.6	10.8 ± 1.6	10.8 ± 1.9	10.8 ± 1.8
Median	12.7	12.4	12.5	10.5	10.7	10.6
Range	8.0–16.0	8.0–17.0	8.0–17.0	7.1–15.9	7.3–16.0	7.1–16.0

### Anatomically preformed screw corridor

The analysis of the anatomically preformed periacetabular screw corridor of the posterior column showed two sites of constriction which are important in daily clinical routine. These constrictions can be found at the inferior margin of acetabulum and at the centre of acetabulum In average, they were located 49.6 ± 3.4 (41.0–60.2) mm (inferior margin of acetabulum) and 77.0 ± 5.6 (66.5–95.3) mm (centre of acetabulum) from the entry point of the implant in men and respectively 43.7 ± 2.3 (38.3–49.3) mm (men vs. women: *p <* 0.001) as well as 71.2 ± 3.5 (63.5–79.9) mm in women (men vs. women: *p <* 0.001). The maximally possible implant diameter at the first constriction (inferior margin of acetabulum) was 15.6 ± 2.3 (10.0–21.0) mm in men and 12.7 ± 2.2 (8.0–18.0) mm in women (men vs. women: *p <* 0.001); at the centre of acetabulum, the second constriction, maximum implant diameter was 15.2 ± 2.1 (10.0–20.0) mm in male patients versus 13.4 ± 1.9 (8.0–17.0) mm in female patients (men vs. women: *p <* 0.001) (Tables 5 and 6). With an average implant length of 141.8 ± 7.7 (125.8–162.0) mm in male patients respectively 127.2 ± 6.8 (111.7–145.3) mm in female patients these constrictions are reached after 35 % and 54 % (men) respectively 34 % and 56 % (women) of the screw length.

**Table 5:** First constriction at the inferior margin of acetabulum. In retrograde posterior column screw placement, first constriction of the anatomically preformed bone corridor could be found at the inferior margin of acetabulum in examined Europeans with uninjured pelvis

	Constriction inferior margin of acetabulum			
	Distance from entry point	Max. implant diameter	Medio-lateral diameter	Sup.-inf. diameter
Men:				
Number	130	130	130	130
Mean ± SD	49.6 ± 3.4	15.6 ± 2.3	19.9 ± 3.5	32.3 ± 3.6
Median	49.1	16.0	19.6	32.4
Range	41.0–60.2	10.0–21.0	13.5–51.3	22.3–41.2
Women:				
Number	130	130	130	130
Mean ± SD	43.7 ± 2.3	12.7 ± 2.2	16.4 ± 2.1	28.7 ± 3.7
Median	43.6	12.0	16.5	28.7
Range	38.3–49.3	8.0–18.0	10.5–21.9	19.8–37.6

**Table 6:** Second constriction at the acetabulum centre. In retrograde posterior column screw placement, second constriction of the anatomically preformed bone corridor could be found at the centre of acetabulum in examined Europeans with uninjured pelvis

	Constriction at the acetabulum centre			
	Distance from entry point	Max. implant diameter	Medio-lateral diameter	Sup.-inf. diameter
Men:				
Number	130	130	130	130
Mean ± SD	77.0 ± 5.6	15.2 ± 2.1	30.0 ± 3.9	20.8 ± 2.8
Median	76.8	15.0	30.4	21.0
Range	66.5–95.3	10.0–20.0	20.1–39.5	13.4–28.2
Women:				
Number	130	130	130	130
Mean ± SD	71.2 ± 3.5	13.4 ± 1.9	24.8 ± 2.8	18.3 ± 2.4
Median	70.8	13.5	25.0	18.1
Range	63.5–79.9	8.0–17.0	11.2–30.4	11.2–23.5

**Fig. 4:**
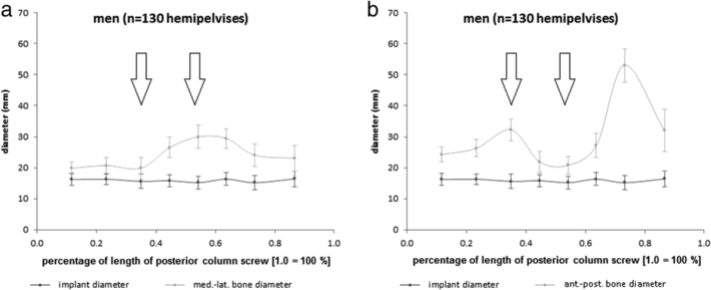
Bone corridor configuration of the posterior column in male patients. **a** Medio-lateral and (**b**) superior-inferior diameter of the bone stock in relation to the maximally possible implant diameter. Important constrictions (marked with arrows) for daily clinical routine are the inferior margin of acetabulum as well as the acetabular centre. (Scaling of x-axis: *0.0* = anterior column at the entry point of the implant; *1.0* = anterior column at the end point of the implant; *n =* 130 hemipelvises)

**Fig. 5:**
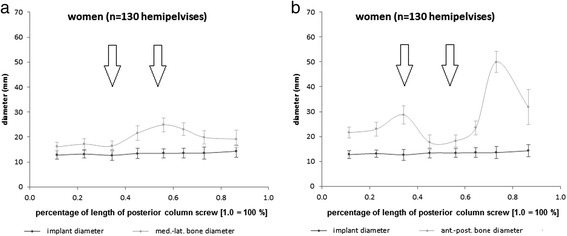
Bone corridor configuration of the posterior column in female patients. **a** Medio-lateral and (**b**) superior-inferior diameter of the bone stock in relation to the maximally possible implant diameter. Important constrictions (marked with arrows) for daily clinical routine are the inferior margin of acetabulum as well as the acetabular centre. (Scaling of x-axis: *0.0* = anterior column at the entry point of the implant; *1.0* = anterior column at the end point of the implant; *n =* 130 hemipelvises)

The detailed configuration of the anatomically preformed bone corridor for the posterior column screw in respective to the medio-lateral and superior-inferior diameter of the bone stock in relation to the maximum screw diameter is shown in Figs. 4 and 5 for male respectively female patients.

Statistical analysis showed significantly higher medio-lateral (*p <* 0.001) and superior-inferior (p ≤ 0.001) bone stock diameters in men compared to women. According to that, the maximally possible screw diameter was higher in male than in female patients at the first (*p <* 0.001) and second constriction (*p <* 0.001).

### Angles between screw trajectory and anterior pelvic plane as well midsagittal pelvic plane and correlation of screw trajectory and anthropometric parameters

The angle between the screw trajectory and the anterior pelvic plane in sagittal section was 14.0 ± 4.9 (2.5–28.6) °, a significant difference was found between both sexes (men vs. women *p <* 0.001). The angle between the screw trajectory and the midsagittal plane in axial section was 31.1 ± 12.8 (1.5–77.9) ° with significant difference between men and women (*p <* 0.001). The angle between the screw trajectory and the midsagittal plane in coronal section was 8.4 ± 3.8 (1.5–20.0) ° also with a significant difference between both sexes (*p <* 0.001). Details are shown in Table 7.

**Table 7:** Angles (in degree) between screw trajectory and anterior pelvic as well midsagittal pelvic plane. Angles in degree between screw trajectory and anterior pelvic as well midsagittal pelvic plane in different sections

	Angle screw trajectory/anterior pelvic plane in sagittal section (°)	Angle screw trajectory/midsagittal plane in axial section (°)	Angle screw trajectory/midsagittal plane in coronal section (°)
Patient collective:			
Number hemipelvises	260	260	260
Mean ± SD	14.0 ± 4.9	31.1 ± 12.8	8.4 ± 3.6
Median	13.7	31.0	8.0
Range	2.5–28.6	1.5–77.9	1.5–20.0
Men:			
Number hemipelvises	130	130	130
Mean ± SD	12.9 ± 4.4	37.3 ± 11.0	9.5 ± 6.3
Median	12.7	36.1	9.3
Range	2.5–22.6	15.4–77.9	1.9–19.8
Women:			
Number hemipelvises	130	130	130
Mean ± SD	15.0 ± 5.0	24.9 ± 11.3	7.2 ± 3.6
Median	14.2	26.9	6.9
Range	4.5–28.6	1.5–55.7	1.5–20.0

## Discussion

Percutaneous stabilization as a minimally invasive treatment option is possible with non-displaced but instable fractures of the acetabulum with a risk of secondary displacement to allow early mobilization of the patient [10, 11]. Moreover, displaced acetabular fractures which can be reduced via lag screw are suitable for percutaneous stabilization [3]. Patients with an elevated perioperative risk from an open approach such as old patients or patients with several injuries in which a reduced approach related morbidity outweighs perfect anatomic fracture reduction, should also be considered for percutaneous stabilization. In addition to this, patients who will need total hip arthroplasty in the short run due to the configuration of the fracture might also benefit from earlier mobilization and protection of soft tissue when undergoing percutaneous stabilization instead of open reduction.

Percutaneous screw placement in the posterior column is difficult due to the complex three-dimensional pelvic geometry. Despite improved imaging with new fluoroscopic views of the acetabulum, 3D-CT scans and the intraoperative use of computer-assisted navigation, there is a danger of perforation the bone corridor or the acetabulum [2, 12]. Therefore, it is important that the surgeon visualizes the bone stock with the screw corridor in preoperative planning.

The present work defined reproducible screw positions in order to analyse the narrow bone corridor of the posterior column. The study was performed on CT-scans of 130 uninjured Europeans as described by [2]. The advantage of this method used in the present work is the fact, that all screws were virtually planned using advanced 3D planning software which is used in daily clinical routine, especially in neuro—and craniofacial surgery (iPlan® CMF 3.0, BrainLAB AG, Feldkirchen, Germany). One of the main focuses of the study was to investigate gender-specific differences in minimally invasive posterior column screw placement, especially in regard to reference planes which are clinically used in pelvic surgery. So far, there is only one study with this focus [3].

The present study showed that mean implant length in men was 141.8 mm and 127.2 mm in women with significant difference between men and women (*p <* 0.001). Shahulhameed et al. in 2010 found a mean posterior column screw length of 138.5 mm for the whole collective of 11 pairs of adult cadaveric pelves; this is in accordance to our findings [8]. Mu et al. however found a mean screw length of 104.8 mm for the whole collective of 30 adult dried hemipelvis specimens [7]. The difference in screw length can be attributed to the fact, that Mu et al. placed antegrade screw whereas we used retrogradely placed screws in the posterior column. The percutaneous minimally invasive approach only allows for retrograde screw placement. We deliberately chose this approach since it offers the advantage of highly reduced soft tissue trauma compared to open approaches [1].

The entry point of the posterior column screw was located at mean 12.7 mm dorsal from the transition of ramus pubis inferior and ischial tuberosity in anterior-posterior direction and at mean 12.1 mm lateral of medial margin of ischial tuberosity in male patients. In female patients, the entry point was located significantly more medial than in male patients (mean: 10.8 mm). Similar gender-specific differences of the entry point could be found by Puchwein et al. [13] and by Dienstknecht et al. [3]. In another study, Dienstknecht also found a gender-specific entry point in posterior column screw placement when measuring the distance from the screw entry point to the landmarks ischial tuberosity, centre of symphysis, iliopectinal eminence and anterior inferior iliac spine [14].

The analysis of the anatomically preformed bone corridor of the posterior column was virtually performed using axial sections referenced to the screw axis according to the method described by Ebraheim et al. [6]. In order to account for the individual anatomy, the axial sections were adjusted to anatomical landmarks such as the walls of the acetabulum. Other workgroups did not take individual anatomical characteristics into account but used sections in defined distances along the screw axis [3, 5, 6]. Thus, in our study, two constrictions of crucial clinical importance in the bone stock of the anterior column could be identified. These constrictions were located at mean 49.6 mm (inferior margin of acetabulum) and 77.0 mm (centre of acetabulum) from the entry point of the implant in men and respectively 43.7 mm (men vs. women: *p <* 0.001) as well as 71.2 mm in women. Puchwein et al. analysed 50 polytrauma patients. Here, the mean distance between the narrowest zone and entry point was 54.8 mm (men) respectively 49.4 mm (women) [13]. The different findings in this study compared to the present study can be attributed to different measurement algorithms: To achieve interindividual comparability, in the present study, defined sectors along the screw axis according to the method described by Ebraheim et al. [6] were used, whereas Dienstknecht et al. measured the narrowest acetabular zone along the screw axis [3].

In our collective, the maximally possible implant diameter was at mean 12.9 mm in men and 11.2 mm in women. This means that the established use of 7.3 mm screws in men and women would have been save. Similar results were found by Banerjee in 2011 [15] and by Shahulhameed et al. in 2010 [8]. It is also in accordance to Attias et al. [5], Puchwein et al. [13] as well as Dienstknecht et al. [3].

As mentioned above we wanted to evolve from single osseous landmarks to established reference planes which can easily be identified intraoperatively; this is why we have chosen the anterior pelvic plane and the midsagittal plane as reference plane and measured the angles between these planes in different CT scans. The anterior pelvic plane [9] and the midsagittal plane are well known from hip arthroplasty [16–21] and are commonly used and easily visualized in the OR. We measured the angle between the posterior column screw as well as the midsagittal plane and the anterior pelvic plane for each hemipelvis comparing the angles found in male and female patients.

The mean angle between the screw trajectory and the anterior pelvic plane in sagittal section was 14.0 °, the mean angle between the screw trajectory and the midsagittal plane in axial section was 31.1 ° and the mean angle between the screw trajectory and the midsagittal plane in coronal section was 8.4 °. For all angles, significant differences were found between both sexes (men vs. women *p <* 0.001).

Dienstknecht et al. also measured angles formed between the posterior column screw and several references lines and two different planes on a.p. radiographs. These reference lines were the tangent to the pubic tubercule, the caudal border of superior pubic ramus, the linea ilioischiadica and the tangent to acetabular rim. A defined vertical and horizontal plane were used as reference planes to measure the angles [3]; this approach has some limitations: The measurement was performed on standard a.p. radiographs, thus only in a two dimensional system compared to the three-dimensional CT scans used in the present study. Moreover, the planes chosen were defined as vertical and horizontal and are not further described. Even more important with respect to the used a.p. radiographs are the findings of Pullen et al. who could show in 2014 that the individual positional variability in pelvic standard radiographs can lead to large-magnitude changes in radiographic acetabular measures [22]. The present study eliminates these problems, since CT scans were used and the reference planes were defined by the pelvic bone landmarks and individually adopted for each pelvis examined. Moreover, the used planes are well established in daily clinical routine and are easily visualized both during procedure planning and in the OR.

However, there are limitations to the present study. The findings are limited to retrograde lag screw placement in the posterior acetabular column and the study has been performed in healthy individuals with no fracture present. Moreover, only European patients were examined, so the findings may be limited for use in Europeans. The effectiveness of the described technique in regard to safe screw placement has to be proved in clinical use.

## Conclusions

The anatomical differences in the pelvic geometry of men and women lead to gender-specific differences in preformed bone corridor, implant length and diameter as well as angles formed between screw trajectory of posterior acetabular column screw and the reference planes (anterior pelvic and midsagittal plane). The angles and the reference planes are reproducible and can be easily visualized in the OR, so they can be helpful in minimally invasive retrograde screw placement in the posterior column. The effectiveness of the described technique in regard to safe screw placement has to be proved in clinical use.
